# Feasibility of Three Novel Forms of Passive Exercise in a Multisensory Environment in Vulnerable Institutionalized Older Adults with Dementia

**DOI:** 10.3233/JAD-190309

**Published:** 2019-08-03

**Authors:** Marelle Heesterbeek, Eddy Anton van der Zee, Marieke Joan Gerda van Heuvelen

**Affiliations:** a Molecular Neurobiology, Groningen Institute for Evolutionary Life Sciences (GELIFES), University of Groningen, Groningen, The Netherlands; b Center for Human Movement Sciences, University of Groningen, University Medical Center Groningen, Groningen, The Netherlands

**Keywords:** Dementia, exercise therapy, feasibility studies, long-term care, nursing home, randomized controlled trial, sensory art therapies, vulnerable populations

## Abstract

**Background:**

Increasing physical activity levels in patients with dementia can reduce pathology severity and progression of the disease. However, physical activity programs can be challenging to adhere to for this vulnerable population. Three novel forms of passive exercise in a multisensory environment may be feasible alternatives for patients who can no longer be involved in physical activity.

**Objective:**

To determine the feasibility of three different forms of passive exercise in a multisensory environment in inactive institutionalized older adults with dementia.

**Methods:**

120 patients with dementia participated in this single blind randomized controlled trial (64.5% female, age 85.3±6.8 years Mini-Mental State Examination range 0–29). Ninety participants were randomly assigned to one of the three intervention groups: Therapeutic Motion Simulation (TMSim), Whole Body Vibration (WBV), and TMSim + WBV. Participants received 6 weeks of passive exercise, 4 sessions a week, 4 (WBV) to 12 (TMSim and TMSim + WBV) minutes per session. Feasibility of the novel forms of passive exercise was evaluated based on attendance, compliance, (proxy) experience scores, adverse events and drop-out rates.

**Results:**

On average 87.9% of the offered intervention sessions were attended. All three forms of passive exercise were well appreciated by the participants (7.3 on a scale from 0 to 10). Intervention related drop-out rates were reasonable (12.2%) and no serious adverse events occurred.

**Conclusion:**

The novel passive exercise interventions TMSim, WBV, and TMSim + WBV are feasible to apply in patients at all stages of dementia. More research is needed to establish effectiveness of passive exercise to limit adverse effects of dementia.

## INTRODUCTION

Older adults with dementia frequently have low activity levels, poor mobility, and reduced quality of life (QoL), especially after being institutionalized. Compared to healthy older adults, community dwelling dementia patients have 21.6% lower physical activity (PA) levels and for institutionalized dementia patients PA levels are even 40% lower [1, 2]. Increasing PA levels by means of exercise interventions can reduce pathology severity and decline in cognitive functioning, QoL, and activities of daily life (ADLs) in patients with dementia [3, 4].

Although increased PA can be effective to reduce disease progression [3–5], adherence to PA programs can be challenging, especially for institutionalized older adults with dementia. Reduced cognitive and physical functions, poor mobility, and comorbidities limit patients in the ability to successfully adhere to or complete PA interventions [6, 7]. In addition, patients who show more severe disease progression are often not even included in studies in which feasibility and effectiveness of new PA programs are tested and evaluated [4]. This makes outcomes of such PA interventions less generalizable to patients in the most severe stages of dementia. Moreover, despite the growing evidence of the benefits of PA in dementia, there remains a lack of studies looking into activity alternatives suitable for patients who are no longer able to stay involved in PA. Therefore, alternatives to PA that are available for all dementia patients, regardless of their cognitive and physical abilities, could be promising in this vulnerable population.

**Fig.1:**
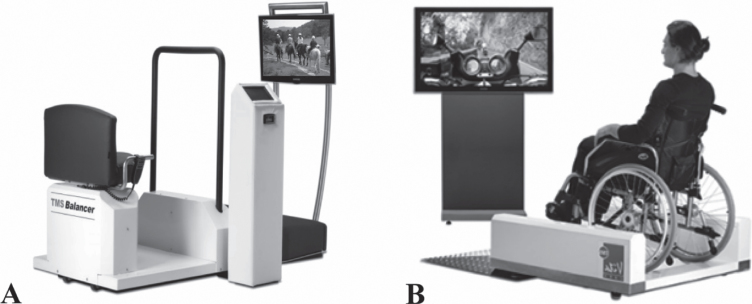
A) The balancer with a chair, screen, and control panel, and B) the wheelchair pod with a wheelchair platform and television screen (identical control panel as in Fig. 1A is not depicted). A wheelchair as well as a normal chair could be safely secured on the wheelchair platform. Both platforms are used to provide the TMSim, WBV, and TMSim + WBV intervention sessions.

We aim to test the feasibility of three new forms of passive exercise in a multisensory environment which are thought to be applicable in all dementia stages. The three novel forms of passive exercise that are distinguished are Therapeutic Motion Simulation (TMSim), Whole Body Vibration (WBV), and the combination of both (TMSim + WBV). All forms of passive exercise are employed with robotized movement platforms as depicted in Fig. 1, making it possible to include wheelchair bound patients as well. During TMSim, tactile, proprioceptive, auditory, and visual stimuli are provided to the user by means of activity videos that are accompanied by matching music and sounds and movements of the platform that are synchronized with the activities on the screen. During WBV, tactile and proprioceptive stimulation is provided to the user via mechanical vibrations (30 Hz, 1–2 mm) of the platform.

WBV exercise, where participants have to performs exercise on a vibrating platform, has been shown to be an effective stimulus for creating significant improvements in overall health [8, 9]. In addition, it has been shown that passive WBV, where participants are seated on a vibrating platform, can improve aspects of attention in (older) adults [10–12]. However, passive WBV has not yet been studied in patients with dementia, nor in combination with other sources of sensory stimulation. The same holds true for TMSim, which has never been studied before. However, after interventions for patients with dementia in which components of TMSim were used, such as music and video interventions, agitated behavior was reduced and social behavior, alertness, and happiness were promoted [13–15]. Taken together TMSim, WBV and TMSim + WBV are thought to help to reduce inactivity in patients with dementia and to be applicable in in all dementia stages. In addition, these novel forms of passive exercise could potentially enhance physical, cognitive, and emotional function in patients with dementia.

WBV was already found to be feasible to apply in institutionalized older adults [16]. However, to the best of our knowledge, feasibility of WBV as well as completely novel interventions such as TMSim and TMSim + WBV are currently not used in older adults with dementia and its feasibility needs to be established. Therefore, the objective of this study was to determine the feasibility of three different forms of passive exercise in a multisensory environment (TMSim, WBV, and TMSim + WBV) in inactive institutionalized older adults with dementia.

## METHODS

The study design was a single blind randomized controlled trial. The study protocol conforms to the principles of the Declaration of Helsinki and was approved by the medical ethics committee of the University Medical Center Groningen (the Netherlands). A detailed description of study design and procedures can be found in the protocol paper of this study [17]. Procedures and methodology relevant to the current paper are described below.

### Participants and procedures

120 residents (64.5% female, age 85.3±6.8 years) from the closed wards of eight different nursing homes in the North of the Netherlands participated in this study. The patient and legal representative were informed about the study and asked to give informed consent. The legal representative gave written informed consent and the patient orally agreed to take part in the study. After informed consent was given, participants were screened for inclusion and exclusion criteria. Participants were enrolled in the study if they were 1) officially diagnosed with a form of dementia, 2) over 65 years of age, and 3) not physically active for more than 10 minutes a day. Participants were excluded if they 1) had a contra-indication for passive exercise, 2) had a serious auditory disorder, 3) were color blind, and/or 4) excessively used alcohol or drugs.

After stratification for nursing home, age, gender, and baseline Mini-Mental State Examination (MMSE) score, participants were randomly assigned to one of the intervention groups or the control group with a 1:1:1:1 allocation ratio. An independent blinded researcher, not related to the study, performed the randomization using a random number generator.

### Interventions

Participants in the intervention groups received 4 (WBV) or 12 (TMSim and TMSim + WBV) minutes of passive exercise in a multisensory environment, four times a week for six consecutive weeks. Participants in the control group received regular care during these six weeks. All forms of passive exercise were applied using two commercially available motion simulation devices as shown in Fig. 1 (*balancer* and *wheelchair pod*, Pactive Motion, Hoogerheide, The Netherlands). During the sessions, participants were asked to take place on either one of the platforms and focus on the television screen. Hands were placed on the sidebars of the balancer or the wheelchair. Preferably the participant was seated as upright as possible. All sessions were individually supervised by a trained research assistant.

In the TMSim intervention group, participants saw three short, real life movies of approximately four minutes each during each session. Matching music and sounds were played and the platform moved synchronically with the movies on screen, so the participants were passively moved and were stimulated multisensory by means of visual, auditory, tactile, and proprioceptive stimuli.

During a WBV intervention session, participants received vibrations with a frequency of 30 Hz with an amplitude of 1-2 mm for four minutes. A stationary motorcycle with idling engine was shown on the screen and matching sounds were played. Three different stationary motorcycling videos were available to provide some sort of variation.

In the TMSim + WBV intervention, the former two forms of passive exercise were combined. During 12 minutes, the participants alternately received TMSim (4 minutes) and WBV (2 minutes).

For the TMSim parts of the intervention a total of 31 different movies were available in six different categories: motor riding, horse riding, nature (e.g., diving, forest, and seaside videos), walking, dancing, and extreme sports (snowboard and jet ski). During the first week, movies from all six categories were shown and participants were asked to give an indication of which movies they liked most. For each session of TMSim and TMSim + WBV, respectively, a top 3 and top 2 were documented. Since not every participant was able to express their preferences also a proxy top 3 and top 2 were given by the research assistant who supervised the session. Selection of movies during the following five weeks was based on the preferences as expressed by the participant after all prior sessions.

In addition, after each session participants were asked to give an indication of how much they enjoyed the session. This was documented as the experience score. Scores could range between zero (not liking the session at all) and ten (really enjoyed the session). As not every participant was able to understand or formulate an experience score, the research assistant scored a proxy-experience score first and thereafter asked the participant to give an experience score to the session (if possible).

### Feasibility

The feasibility of TMSim, WBV, and TMSim + WBV in institutionalized older adults was evaluated based on the following parameters: attendance, compliance, (proxy) experience scores, adverse events, and drop-out rates. Attendance was computed after the 6-week intervention period as the percentage of the offered sessions that were actually attended. Compliance was the percentage of attended sessions that were completed according to protocol. Experience scores, between 0 (very unpleasant) and 10 (very pleasant), were obtained for each completed session [17]. The following ranges apply to the experience scores: 0–3 very unpleasant, 3–5 unpleasant, 5–6.5 neutral, 6.5–8 pleasant, 8–10 very pleasant. Adverse events, drop-outs, and reasons for drop-out were documented. A distinction was made between dropouts related to the intervention (e.g., unwillingness to participate after a number of sessions or drop-outs related to adverse events) and drop-outs not related to the intervention (e.g., sickness and death).

An intervention was considered as feasible when attendance was high (>75%), interventions were completed according to protocol (>90%), experience scores indicated at least pleasant experiences (≥6.5) for the participants, drop-out rates related to the intervention were low (<20%), and no serious adverse events occurred [18].

### Statistics

**Fig.2:**
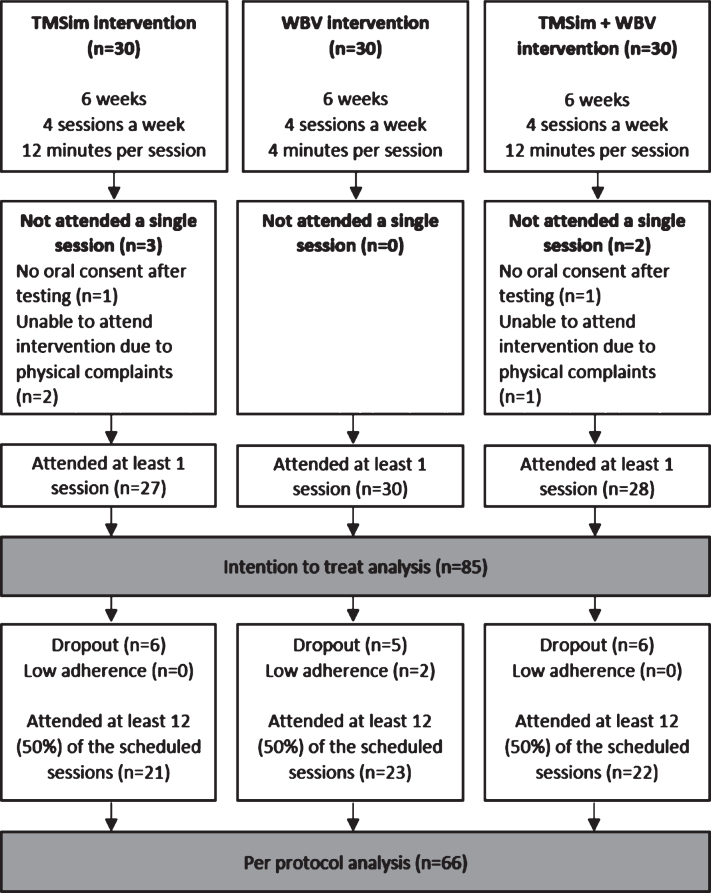
A flowchart of the participants per group included in the intention to treat and the per protocol analyses.

Data was analyzed using IBM SPSS statistics 25. Both intention-to-treat as well as per protocol analyses were performed. In the intention to treat analysis, every randomized participant who participated in at least one session was included. For the per protocol analysis, only participants who attended at least 50% of the scheduled sessions were included. A flowchart for the groups and participants included in the intention to treat and per protocol analyses is presented in Fig. 2. Chi-square tests were used to analyze differences in gender, use of walking aid/wheelchair, type of dementia, platform use, and dropout rates between the groups. Age, number of comorbidities, MMSE score, and intervention related measures were tested using one-way analysis of variance. Bonferroni corrected *post-hoc* tests were performed for significant group effects. *p* values lower than 5% were considered to indicate statistical significance.

## RESULTS

### Group characteristics

In Table 1, baseline characteristics of the 120 included participants are presented. Age, gender, use of walking aid, number of comorbidities, and global cognitive function as measured with the MMSE were not different at baseline between the four groups.

**Table 1:** General characteristics

Characteristic	TMSim group (*n* = 30)	WBV group (*n* = 30)	TMSim + WBV Group (*n* = 30)	Control group (*n* = 30)	*F/χ*^2^ test-*value (df), p*
Age (y), M(SD)	84.9(6.6)	86.2 (4.7)	84.3(8.1)	85.8(7.4)	0.48 (119), *p* = 0.70^a^
Range	69–95	75–96	69–103	70–99
Females, %	70.0	63.3	66.7	66.7	0.30 (3), *p* = 0.96^b^
Walking aid/wheelchair	11/6	13/4	15/3	11/8	3.91 (6), *p* = 0.69^b^
Dementia type^c^, %					10.1 (15), *p* = 0.81^b^
*Alzheimer*’*s disease*	53.3	43.3	60	36.7
*Vascular dementia*	10	16.7	13.3	13.3
*Lewy body dementia*	3.3	3.3	0	0
*Frontotemporal dementia*	3.3	0	0	3.3
*Combination*	10	13.3	3.3	10
*Other/Unknown*	20	23.3	23.3	36.7
Comorbidities (number), M±SD, N	2.8±1.8, (26)	3.6±2.0, (21)	3.4±2.0, (16)	3.7±2.7, (23)	0.90 (85), *p* = 0.44^a^
MMSE^d^, M(SD)	12.2(7.5)	14(5.9)	13.6(6.7)	12.8(6.1)	0.38 (98), *p* = 0.77^a^
quad Range^d^	0–29	5–22	3–28	1–26
*N/not tested*	23/7	26/4	27/3	23/7
*Not tested due to (receptive) aphasia/unwillingness*	4/3	1/3	2/1	3/4
*Questionable (score 25*–*30), %*	4.3	0	3.7	4.3
*Mildly impaired (score 19*–*24), %*	17.4	26.9	25.9	13.1
*Moderately impaired (score 10*–*18), %*	39.2	42.3	44.5	56.5
*Severely impaired (score* ≤*9), %*	39.1	30.8	29.6	26.1

^a^Differences between groups were tested with One-way Analysis of Variance. ^b^Differences between groups were tested with *χ*^2^ test. ^c^Presumed dementia type according to medical records. ^d^Mini-Mental State Examination. Range 0–30, higher scores indicate better performance.

### Personalization

**Fig.3:**
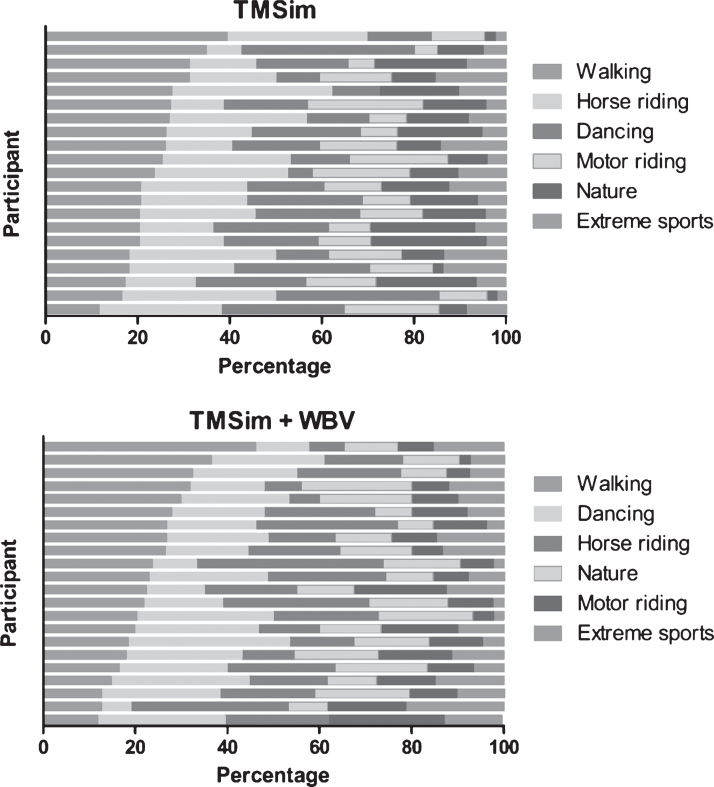
Overview of the percentages of the videos that are played in each category in the TMSim and TMSim + WBV group. For each group the categories are sorted (descending) based on the total number of videos watched in each category. Each bar represents a single individual. Only individuals who attended at least 50% of the scheduled sessions were included in this figure.

In Fig. 3, the percentage of videos watched per category in the TMSim and the TMSim + WBV group are presented for each individual. In general, walking was the most often watched category in both the TMSim and the TMSim + WBV group. In both groups, the videos least watched were in the category extreme sports. However, large variation in preferred categories can be observed between individuals in both the TMSim as well as the TMSim + WBV group.

### Adverse events and drop-outs

No serious adverse events took place in relation to the different passive exercise interventions. A total of four participants experienced motion sickness during the intervention session and therefore further interventions were stopped for these participants. However, it must be noted that two of these patients also reported motion sickness when movements of the device were turned off.

Drop-out rates did not significantly differ between the groups. In addition to the drop-outs due to motion sickness, a total of two participants passed away during the study, and due to illness (e.g., broken shoulder, pneumonia), eight patients were no longer able to attend the intervention sessions. After a couple of sessions, a total of seven participants indicated that they did no longer want to participate in the study. Eleven drop-outs were related to the intervention. In the Supplementary Material, the number of dropouts is given with regard to physical and cognitive burden of the participants. No apparent differences in drop-out rates were observed with regard to physical and cognitive disease severity of the participants.

### Intention to treat

In Table 2, feasibility measures for each group are presented. 85 of the 90 participants that were assigned to one of the intervention groups were included in the intention to treat analysis. Five participants did not attend a single intervention session; hence these participants were not included in the analysis. On average, 20.4±6.9 passive exercise sessions were offered to the participants of which 16.6±7.8 were attended. Reasons for missed sessions were divers: motivational problems (40%), bedday (23%), sick (9.1%), not present at ward (7.9%), visitors (5.5%), delusions (3.9%), physical discomfort (3.3%), tiredness (3.3%), aggressive behavior (2.1%), discommended by staff (1.2%), disoriented (0.3%), and broken wheelchair (0.3%). Mean attendance rates ranged from 77% (TMSim and TMSim + WBV) to 83% (WBV). Overall compliance of the attended sessions was 99.3%, cases of non-compliance were caused by participants wanting to leave the session before it was finished.

**Table 2:** Feasibility outcome measures presented per group

Intervention characteristic	TMSim group	WBV group	TMSim + WBV group	Control group	*F/χ*^2^ test-value (df), p
Balancer/wheelchair platform (N)	17/13	17/13	18/12	N.A.	0.09 (2), *p* = 0.96^b^
Intention-to-treat analysis*, N*	27	30	28	N.A.
*Sessions offered, M(SD)*	20.1(7.0)	20.9(6.9)	20.3(6.9)	N.A.	0.12 (85), *p* = 0.88^a^
*Sessions attended, M(SD)*	16.3(8.2)	17.4(7.7)	16.1(7.7)	N.A.	0.24 (85), *p* = 0.79^a^
*Attendance rate*^c^*, M(SD) %*	77.2(26.3)	82.5(22.0)	77.1(27.5)	N.A.	0.44 (85), *p* = 0.65^a^
*Compliance, %*	100	99.8	98.0	N.A.
*Experience, M(SD)* *N*	7.2(1.3) 17	6.9(1.7) 22	7.3(0.9) 20	N.A.	0.54 (58), *p* = 0.59^a^
*Proxy experience, M(SD)*	7.1(0.9)	6.3(0.8)	6.7(0.7)	N.A.	7.68 (77), *p* = 0.001^a^
Per protocol analysis, *N*	21	23	22	N.A.
*Sessions offered, M(SD)*	23.2(1.2)	23.7(0.9)	23.1(1.1)	N.A.	2.28 (65), *p* = 0.11^a^
*Sessions attended, M(SD)*	20.8(2.5)	21.3(2.9)	19.5(4.0)	N.A.	1.80 (65), *p* = 0.17^a^
*Attendance rate*^c^*, M(SD) %*	89.6(11.4)	89.7(12.5)	84.5(17.3)	N.A.	0.98 (65), *p* = 0.38^a^
*Compliance, %*	100	99.8	97.9	N.A.
*Experience, M(SD)*	7.4(1.3) 14	7.4(0.9) 19	7.2(0.9) 19	N.A.	0.15 (51), *p* = 0.87^a^
*N*	14	19	19
*Proxy experience, M(SD)*	7.2 (0.9)	6.5 (0.5)	6.7 (0.7)	N.A.	6.49 (65), *p* = 0.003^a^
Drop-out, N	8	5	9	1	13.3 (15), *p* = 0.58^b^
*No oral consent after testing*	1^d^	0	1^d^	N.A.
*Motion sickness*	1	1	2	N.A.
*Refused to attend after 1*–*16 sessions*	2	2	3	N.A.
*Illness/Physical complaints*	3^d^	2	3^d^	0
*Passed away*	1	0	0	1

N.A., not applicable. ^a^Differences between groups were tested with One-way Analysis of Variance. ^b^Differences between groups were tested with *χ*^2^ test. ^c^The percentage of offered sessions that are attended. Note that if scheduled sessions would have been used attendance rates would have been lower. ^d^Includes participants who did not attend a single session.

Fifty-nine participants were able to express their experiences. The passive exercise sessions on average were rated between 6.9 (WBV) and 7.3 (TMSim + WBV) by the participants with no significant differences between groups. Proxy-experience scored by the supervisor of the sessions was significantly different between groups, *post hoc* analyses revealed the proxy-experience was significantly higher in the TMSim group when compared to the WBV group (*p* = 0.004; 95% [CI], 0.23 to 1.47).

### Per protocol

Due to a variety of setbacks (e.g., closing of ward due to norovirus, technical problems, or sickness of the research assistant) for 65% of the participants the intended 24 sessions were offered, 12% were offered 23 interventions sessions, and 15% were offered 22, 6% were offered 21 sessions, and a final 1.5% was offered 20 sessions. Of the 90 participants that were assigned to an intervention group, 68 participants attended at least 12 (50%) of the scheduled sessions. Mean adherence rates ranged between 85% (TMSim + WBV) and 90% (TMSim and WBV). Overall 99.3% of the attended sessions in the per protocol analysis was completed according to protocol. In general, the experience and proxy-experience scores were somewhat higher in the per protocol analyses as compared to the intention-to-treat analyses, but these differences were not significant (*p* > 0.05). Overall 52 participants were able to judge the sessions and indicated that they experienced the passive exercise sessions as pleasant (6.5–8). Experience scores were highest in the TMSim group, followed by WBV and TMSim + WBV. The TMSim sessions also tended to be rated better by the supervisor as compared to the WBV and the TMSim + WBV sessions. Proxy experience scores were significantly higher in the TMSim group when compared to the WBV group (*p* = 0.002; 95% [CI], 0.24 to 1.32).

## DISCUSSION

The objective of this study was to evaluate the feasibility of three new forms of passive exercise, TMSim, WBV, and TMSim + WBV, in institutionalized older adults with dementia. The interventions were considered feasible when attendance and compliance were high (respectively, >75% and >90%), experience scores indicated at least pleasant (≥6.5) experiences for the participants, drop-out rates related to the intervention were low (<20%) and no serious adverse events occurred.

All three forms of passive exercise in a multisensory environment were successfully delivered over a 6-week time period, and even the most severely affected individuals could successfully adhere to the program. The results indicate that all three forms of passive exercise in a multisensory environment were well appreciated by the participants (mean experience 7.3, range 7.2–7.4). Moreover, attendance rates are considered as high (mean attendance 87.9%, range 84.5–89.7), interventions were completed according to protocol in 99.3% of the sessions, intervention related drop-out rates were reasonable (12.2%, range 10.0–17.9) and no serious adverse events occurred. All things considered, TMSim, WBV, and TMSim + WBV are feasible to apply in institutionalized older adults with dementia.

Due to the novelty of the described passive exercise interventions, there is limited opportunity to compare feasibility outcome measures with other passive exercise studies. One study in which WBV was applied in nursing home patients (6 weeks, 3 times a week) reported attendance rates over 95% [16]. However, in this study, very strict in- and exclusion criteria were applied with regard to cognitive and physical dysfunction. Since disease severity can affect adherence rates [19], it is likely that differences in attendance rates between these studies are the result of differences in the included sample. Despite the inclusion of even the most severely affected patients, the mean attendance rate of 87.9% is comparable to a study in which feasibility of a group intervention involving multisensory stimulation, reminiscence, and light physical activity in people with moderate to severe dementia (88.6%) [20]. Moreover, mean attendance was 12.8% higher than the mean attendance rate in a 22-week music therapy intervention for care home residents with dementia [21]. As the latter study provided a 22-week instead of 6-week intervention, higher attendance rates could be explained by the shorter intervention period. Longer duration passive exercise programs are needed to establish attendance on the long term.

The high attendance rates we found in this study might also be related to the opportunity to adapt the interventions to the participants’ preferences. We observed large variation in preferences between individuals. As the selected videos were based on participants’ preferences, the variation between individuals represents a certain level of individualization of the TMSim and TMSim + WBV interventions. The possibility to adapt TMSim and TMSim + WBV to participants’ preferences can be considered as a major strength of these interventions. Activities that can be adjusted to people’s preferences and are enjoyable can enhance attendance over the long term [22]. Hence, it is likely that the opportunity to adapt the TMSim and TMSim + WBV interventions to the preferences of the participants has contributed to both high experience scores as well as attendance rates.

Although not significantly different, drop-out rates were higher in the intervention groups when compared to the control group. This can partly be explained by the fact that no intervention was provided to the control group. Therefore, one could only drop-out from the control group if they passed away. Furthermore, for some intervention related drop-outs, the reason for drop-out might not be specific for the intervention given. In a number of cases, the motions of the device were completely turned off, while the participants still indicated they became dizzy. For these dropouts, it can be questioned whether the reported motion sickness was truly an effect of the intervention parameters or more so of the novelty of the intervention and stress that might be related to that.

The overall dropout rate in the intervention groups (24.4%) was relatively high when compared to other studies [16, 21]. This may be explained by the included population. It is very reasonable to assume that with including patients with higher disease severity (e.g., higher level of dependence, cognitive decline) risks for drop-outs not related to the intervention (e.g., death, illness) are higher. In addition, risk for complications during the intervention could be higher as compared to a less severe impaired population. Kovach (2000) described how older adults with dementia can experience intrapsychic discomfort as a result of too little or too much sensory stimulation [23]. This discomfort can result in confusion, agitation, frustration, or unhappiness [24]. For some participants the combinations of sensory stimuli that is provided during passive exercise may have caused sensory overstimulation, resulting in non-attendance or eventually even dropping out. If overstimulation is suspected, parameters of the interventions could be adjusted, even further extending the possibilities to individualize passive exercise.

While for some patients, overstimulation as a result of passive exercise can be a potential threat; for others, passive exercise may propose a great opportunity to breach inactivity patterns and prevent sensory deprivation. Although duration of passive exercise lasted for four to twelve minutes in the current study the duration of passive exercise can easily be extended or shortened, depending on the needs of a patient. As such, passive exercise in a multisensory environment may be used to reduce intrapsychic discomfort in patients with dementia, resulting in less agitation, frustration and confusion and therefore reducing care burden of their caregivers.

One of the limitations of the current study is that the interventions were supervised by trained research assistants and not by nursing home staff. This does not affect the feasibility of the interventions itself, but it does limit us in evaluating the possibility to apply these forms of passive exercise outside the study setting. Future research in which passive exercise is applied by nursing home staff is necessary to test whether passive exercise can be integrated in daily routines within the nursing home setting.

Second, even though the majority of the included participants showed both cognitive and physical disabilities, there might have been an inclusion bias as a result of protectiveness of the legal representatives over potential participants. In the recruitment phase of the study, numerous legal representatives indicated that they thought their relative was too frail to participate in this study and therefore did not provide informed consent. This could have resulted in an included population that was less frail and representative for the vulnerable, inactive population we aimed for. However, the majority of the included participants showed both cognitive and physical disabilities. In addition, there was a substantial number of participants for who assessment with MMSE test was not even possible. Hence, we think the study population does represent the vulnerable population we aimed to include to a large extent.

Taken together, the novel passive exercise interventions, TMSim, WBV, and TMSim + WBV, are feasible to apply in patients at all stages of dementia and can be used to provide passive exercise with (multi)sensory stimulation while respecting people their preferences. The different forms of passive exercise can be used to breach inactivity patterns and might have the potential to reduce mental discomfort in patients with dementia and care burden of their caregivers. More research is needed to establish attendance to passive exercise on the long term and to test whether it is possible to integrate passive exercise in daily routines of the nursing home setting. Moreover, the potential effectiveness of these novel forms of passive exercise in a multisensory environment to limit adverse effects of dementia are worth investigating.

## Supplementary Material

Supplementary Material
