# Supplementary material for: Feasibility of Three Novel Forms of Passive Exercise in a Multisensory Environment in Vulnerable Institutionalized Older Adults with Dementia
Source: J Alzheimers Dis. 2019 Aug 3;70(3):681–90. doi: 10.3233/JAD-190309 (PMC6700638; doi:10.3233/JAD-190309)
Supplement: Supplementary Material [file jad-70-jad190309-s001.docx]

**Supplementary Material**

**Feasibility of Three Novel Forms of Passive Exercise in a Multisensory Environment in Vulnerable Institutionalized Older Adults with Dementia**

**Supplementary Table 1.** Drop-out rates for participants without a walking aid, walking aid users and wheelchair users.

|  | **None** | **Walking aid** | **Wheelchair** |
| --- | --- | --- | --- |
| Drop-out, N/% | 9/18.4% | 10/20% | 4/19% |
| *No oral consent after testing* | 2/4.1% | 0/0% | 0/0% |
| *Motion sickness* | 0/0% | 3/6% | 1/4.8% |
| *Refused to attend after x sessions* | 2/4.1% | 5/10% | 0/0% |
| *Illness/physical complaints* | 4/8.2% | 2/4% | 2/9.5% |
| *Passed away* | 1/2% | 0/0% | 1/4.8% |

**Supplementary Table 2.** Drop-out rates divided by dementia severity level

|  | ***Questionable***  ***(score 25-30)*** | ***Mild dementia (score 19-24)*** | ***Moderate dementia (score 10-18)*** | ***Severe dementia (score ≤ 9)*** | ***Not tested*** ^a^ |
| --- | --- | --- | --- | --- | --- |
| MMSE, N | 3 | 20 | 45 | 31 | 21 |
| Drop-out, N/% | 0 | 3/15.0% | 10/22.2% | 5/16.1% | 5/23.8% |
| *No oral consent after testing* | 0 | 0 | 0 | 0 | 2/9.5% |
| *Motion sickness* | 0 | 0 | 2/4.4% | 2/6.5% | 0 |
| *Refused to attend after 1-16 sessions* | 0 | 1/5.0% | 5/11.1% | 0 | 1/4.8% |
| *Illness/physical complaints* | 0 | 2/10.0% | 2/4.4% | 2/6.5% | 2/9.5% |
| *Passed away* | 0 | 0 | 1/2.2% | 1/3.2% | 0 |

^a^ Mini-Mental State Examination testing was not possible in these participants due to (receptive) aphasia (n=10) or unwillingness to test (n=11)
